# MSC EVs Deliver miRNAs into Cardiomyocytes via EIPA-Sensitive and Clathrin-Mediated Endocytosis

**DOI:** 10.3390/biomedicines14092002

**Published:** 2026-09-05

**Authors:** Chongyu Zhang, Simon Kaja, W. Keith Jones

**Affiliations:** 1 Department of Molecular Pharmacology and Neuroscience, Loyola University Chicago Stritch School of Medicine, Maywood, IL 60153, USA; czhang13@luc.edu (C.Z.); skaja@luc.edu (S.K.); 2 Department of Ophthalmology, Loyola University Chicago Stritch School of Medicine, Maywood, IL 60153, USA

**Keywords:** cardiovascular disease, extracellular vesicles, exosomes, microRNA, endocytosis

## Abstract

**Background/Objectives:** Extracellular vesicles (EVs) have emerged as important mediators of intercellular communication in cardiovascular disease, particularly through the transfer of regulatory microRNAs. However, direct evidence of cytosolic microRNA delivery by mesenchymal stem cell (MSC)-derived EVs into cardiomyocytes remains limited. Many prior studies have inferred transfer without fully excluding endogenous microRNA induction or surface-bound artifacts. The objective of this study was to determine whether MSC-derived EV microRNA undergoes functional cytoplasmic delivery into cardiomyocytes and to identify the endocytic pathways involved. **Methods:** To eliminate background from endogenous microRNA expression, MSC-derived EVs were loaded with the exogenous microRNA cel-miR-39-3p. H9C2 cardiomyocytes were treated with increasing vesicle doses, and intracellular microRNA levels were quantified using real-time polymerase chain reactions. Cytosolic accessibility was assessed indirectly using a cholesterol-modified antagomir targeting cel-miR-39-3p. Pharmacologic inhibitors of macropinocytosis (5-(N-ethyl-N-isopropyl) amiloride (EIPA)) and clathrin-mediated endocytosis (chlorpromazine and ES9-17) were applied to delineate uptake pathways, with transferrin assays confirming clathrin inhibition. **Results:** EV treatment produced a dose-dependent increase in intracellular cel-miR-39-3p levels. Antagomir administration reduced intracellular microRNA levels by 89%, providing indirect functional evidence that transferred cel-miR-39-3p reached an antagomir-accessible cytosolic compartment. At the highest preparation dose, EIPA and ES9-17 reduced cell-associated cel-miR-39-3p by 71% and 36%, respectively, consistent with contributions from EIPA-sensitive and clathrin-associated uptake processes. Notably, chlorpromazine increased microRNA accumulation despite blocking transferrin uptake, suggesting compensatory or membrane curvature-dependent effects. **Conclusions:** These findings provide strong functional evidence that exogenous cel-miR-39-3p associated with MSC EV preparations reaches an antagomir-accessible cytoplasmic compartment in H9C2 cardiomyocytes and implicate EIPA-sensitive uptake consistent with macropinocytosis and clathrin-associated uptake. Additional genetic, localization, and pulse-chase studies are required to establish the precise intracellular trafficking mechanisms. This work strengthens the mechanistic foundation for EV-based therapies and informs the rational development of cell-free strategies for cardiovascular disease.

## 1. Introduction

Extracellular vesicles (EVs) are a heterogeneous group of membranous vesicles secreted by most prokaryotic and eukaryotic cells. Exosomes, microvesicles and apoptotic bodies are the three main types of EVs categorized based on their biogenesis, size, and function by the International Society of Extracellular Vesicles [1,2,3]. Exosomes, approximately 30–150 nm in size, are derived from the late endosomal compartment and secreted through fusion of the multivesicular bodies with the plasma membrane. Microvesicles and apoptotic bodies, often larger in size, are released directly from the cytoplasmic membrane [2]. However, substantial overlap in size and composition between EV subtypes has made their experimental separation challenging, and the recent Minimal Information for Studies of Extracellular Vesicles 2023 (MISEV2023) guidelines emphasize using the general term “EVs” unless biogenesis can be rigorously assigned [3]. Once produced and released from producer cells, EVs can then be taken up by other cells, and their contents are thought to function in the recipient cells. Their contents include lipids, proteins and RNAs. The non-coding RNAs, including long non-coding RNAs (lncRNAs) and microRNAs (miRNAs), can regulate gene product expression in the recipient cells [4,5,6,7,8,9]. Previous work from our lab has demonstrated a cardioprotective role of mesenchymal stem cell (MSC) EVs via transfer of miR-21a-5p [10]. In the same study, MSC EV uptake by H9C2 cells was demonstrated using PKH26 flow cytometry and imaging flow cytometry analysis, as well as fluorescence microscopy of covalent maleimide-Alexa Fluor 488 labeling. We also showed that delivery of this miRNA downregulated apoptotic cell death pathways and decreased infarct size. However, we also pointed out the limitations of that study, including that the cardioprotective effects can be the result of endogenous miR-21a-5p induced by the EV action on recipient cells. In fact, most studies in the EV field assume that the cargo from the EVs is delivered into the recipient cells without direct evidence. After more than a decade of work in this area, there is no rigorous standard to show direct proof of EV miRNA transfer. Following an exhaustive literature search, we have identified 12 published studies to date that provided sufficient evidence of EV-dependent miRNA transfer into recipient cells in different systems, only eight of which demonstrated functional effects of the transferred miRNA (Table 1) [4,11,12,13,14,15,16,17,18,19,20,21]. Some of the studies are representative examples, and additional studies using similar approaches are not listed. Two of these studies concern cardiac cells. Feng et al. utilized a transwell co-culture system using fluorescein-labeled miR-22 mimic transfected into MSCs to generate MSC-derived EVs to test cargo delivery from MSC-derived EVs to neonatal murine cardiomyocytes [14]. Confocal imaging showed fluorescein colocalized with cardiomyocytes, while the parallel quantitative reverse transcription polymerase chain reaction (qRT-PCR) analysis of the cardiomyocytes showed increased miR-22 levels. They also demonstrated that miR-22 functionally repressed *Mecp2* in this system. However, the lack of direct EV treatment of recipient cells, shortcomings of fluorescent imaging, and most importantly the existence of endogenous miR-22 in the recipient cardiomyocytes limit the conclusions. Specifically, qRT-PCR and *Mecp2* repression could be caused by upregulation of the endogenous *miR-22* gene. This illustrates one of the common pitfalls that complicate EV research. In the other study, Ong et al. treated mouse cardiac progenitor cells with EVs from *Caenorhabditis elegans*-miR-39 (cel-miR-39)-transfected mouse endothelial cells (cel-miR-39 does not exist in mammals) [18]. cel-miR-39-3p is not encoded in the mouse or rat genome and therefore provides a sequence-orthogonal tracer with negligible endogenous background in both the donor and recipient cells. The qRT-PCR analysis of the recipient cells showed an increase in cel-miR-39 levels in a time-dependent manner. Using a sequence-orthogonal exogenous miRNA as cargo reduces interference from endogenous miRNA expression in recipient cells. Other non-mammalian miRNAs can be used for the same purpose; for example, cel-miR-54 has been used to assess EV-mediated miRNA transfer in mouse and human recipient cells [19]. However, the drawback is that exogenous miRNAs, especially nematode miRNAs, tend not to have any functional effects in mammalian cells, and their detection alone does not establish localization to a functionally active cellular compartment. In order to study the extent to which EV miRNA is transferred from EVs into recipient cells and whether EV miRNA modulates gene expression levels and cellular function, both aspects have to be rigorously addressed.

Due to their differentiation potential and expansion capacity, mesenchymal stem cells (MSCs) have been studied extensively for therapeutic purposes [8,22]. They are known to carry cardioprotective potential and improve cardiac function following injury [23,24]. Since previous work in our lab and others indicates that the delivery of cardioprotective miRNAs plays a major role in these therapeutic effects [10,25,26,27,28], understanding the mechanisms of MSC EV miRNA delivery in the heart is of great interest for potential therapeutic development of MSC EVs.

It is generally understood that EVs enter cells via endocytic pathways, including clathrin-mediated endocytosis, caveolin-mediated endocytosis, macropinocytosis, phagocytosis, lipid raft-mediated endocytosis and membrane fusion [29,30]. The majority of experimental evidence supports that receptor-mediated endocytosis is a highly specific process that requires matching ligands and receptors from EVs and the recipient cell type. For other processes such as macropinocytosis that do not require contact between the cells and the internalized materials, studies suggest that it is a minor pathway or at least not a universally utilized mechanism for all cell types [29]. Recent work also shows that cardiomyocyte-derived EVs may use alternative transfer routes depending on physiological context such as tunneling nanotubes in the neonatal heart [31]. Therefore, EV uptake is increasingly recognized as a cell-type-specific and receptor-dependent process. When it comes to stem cell EVs in the heart, Eguchi et al. found that hypoxic conditions activate clathrin-mediated endocytosis in cardiomyocytes to take up adipose-derived stem cell EVs [32]. It is important to understand the endocytic mechanism of different EV populations and recipient cell types in each system for translational applications. Herein, we examined miRNA transfer and cytoplasmic accessibility in cardiomyocytes using exogenous cel-miR-39-3p associated with MSC EV preparations.

This study provides functional evidence that cel-miR-39-3p associated with MSC EV preparations reaches an antagomir-accessible cytoplasmic compartment in cardiomyocytes and provides another method to quantitatively investigate EV-associated miRNA transfer. The pharmacologic inhibitor results implicate EIPA-sensitive uptake consistent with macropinocytosis and clathrin-mediated endocytosis in this process. Finally, this study demonstrates that EV-delivered cel-miR-39-3p is susceptible to antagomir-mediated degradation, supporting functional cytoplasmic delivery of EV microRNA cargo [18,19,33]. Taken together with our previous work in vitro and in vivo [10], this work demonstrates that MSC-derived EVs transfer miRNAs into cardiac cells while leaving the exact intracellular trafficking mechanisms to be established.

## 2. Materials and Methods

### 2.1. Cell Culture

Rat-derived H9C2 cardiomyocytic cells (ATCC, Manassas, VA, USA; Cat. No. CRL-1446; mycoplasma contamination not detected) were cultured in Dulbecco’s Modified Eagle Medium (DMEM, Gibco, Thermo Fisher Scientific, Waltham, MA, USA; Cat. No. 11965092), with 10% EV-free fetal bovine serum (Thermo Fisher Scientific, Waltham, MA, USA; Cat. No. 10099158; EV depleted by ultracentrifugation at 100,000× *g* for 90 min, heat-inactivated at 56 °C for 30 min) and L-glutamine (Thermo Fisher Scientific, Waltham, MA, USA; Cat. No. A2916801) supplementation. They were subcultured at a 1:4 ratio in T75 flasks (Thermo Fisher Scientific, Waltham, MA, USA; Cat. No. 156499; MidSci, St. Louis, MO, USA; Cat. No. TP90076). H9C2 cells were selected to maintain continuity with our previous study, which demonstrated uptake and cardioprotective effects of mouse bone marrow MSC EVs in this recipient cell model [10].

### 2.2. Mesenchymal Stem Cell Culture

Frozen stocks of primary murine mesenchymal stem cell (MSC) cultures were used for the experiments presented here. The cultures were originally derived from bone marrow obtained from the tibia and femurs of C57BL6/129SF2/J wild-type mice (Jackson Laboratories, Bar Harbor, ME, USA; stock # 101045) according to the protocol established by Peister et al. [34]. The original animal procedures were approved by the Loyola University Chicago Institutional Animal Care and Use Committee under protocol 206610, “NFκB-dependent miRNAs in cardioprotection and regeneration” (approved on 1 November 2010). No additional live animals were acquired or euthanized, and no newly collected animal tissues were used in the present study. Cells were expanded in Iscove’s Modified Dulbecco’s Medium (IMDM, Gibco, Thermo Fisher Scientific, Waltham, MA, USA), with 10% EV-free fetal bovine serum (Gemini Bio-Products, West Sacramento, CA, USA) and 10% EV-free horse serum (Gibco, Thermo Fisher Scientific, Waltham, MA, USA), penicillin-streptomycin (Thermo Fisher Scientific, Waltham, MA, USA), and L-glutamine (Thermo Fisher Scientific, Waltham, MA, USA) [34]. Cells were plated and washed regularly for 4 weeks, then trypsinized and passed into a new plate as passage 1. Cells were maintained at 37 °C in a humidified incubator with 5% CO_2_. The cells were allowed to grow with media replacement twice per week. Cells were passaged when they reached 80% confluency. Culture was terminated at passage 20 for all subsequent studies. The exact passage used for each EV preparation varied between 5 and 18. The original MSC cultures had previously been characterized by colony-forming unit assays, differentiation cultures, and flow-cytometric cell-surface immunophenotyping [10]. Their immunophenotype was CD29^+^, CD44^+^, Sca1^+^, CD45^−^, and CD11b^−^, which is consistent with the established MSC phenotype [34,35,36].

For EV collection, MSCs remained in the EV-free serum-supplemented IMDM described above; no separate serum-free conditioning interval was used. MSC-conditioned medium generated between the twice-weekly medium changes was collected for EV isolation.

### 2.3. Extracellular Vesicle Isolation

Vesicles were isolated via the method established by Théry et al. [37]. Freshly isolated media was centrifuged at 2000× *g* for 20 min at 4 °C to clear dead cells. Cell membrane-bound fragments were cleared from the resulting isolate by spinning at 10,000× *g* for 30 min at 4 °C. EVs were pelleted via 100,000× *g* for 70 min at 4 °C and resuspended and filtered through a 0.2 µm syringe filter, then ultracentrifuged a second time at 100,000× *g* for 70 min at 4 °C. The EV pellet was resuspended in 200 µL sterile PBS and particle size distribution was analyzed using a NanoSight NS300 instrument with nanoparticle tracking analysis (NTA) software, version 3.4 (Build 3.4.4; Malvern Panalytical Ltd., Malvern, UK), using five approximately 30-s light-scatter recordings (150 s total) per sample. MSC EV preparations generated using this isolation protocol had been characterized previously and were positive for TSG101 and CD9 by immunoblotting [10]. Samples were diluted 20- to 4000-fold in PBS to achieve 25–100 particles per frame and analyzed by nanoparticle tracking analysis using a NanoSight NS300 instrument (Malvern Panalytical Ltd., Malvern, UK) from 150 s light-scatter recordings. NTA-derived particle concentrations of the preparations were approximately 1.0 × 10^11^–10^12^ particles/mL. Total protein concentration was determined using the Protein A280 absorbance application on a BioTek Cytation 5 multimode reader (BioTek Instruments, Winooski, VT, USA). Total protein yield, calculated from the measured concentration and final preparation volume, was 4–10 µg per preparation.

### 2.4. Exo-Fect Loading of Extracellular Vesicles

Extracellular vesicles were loaded using cel-miR-39-3p mimic in full batches for each experiment using Exo-Fect siRNA/miRNA Transfection Kit (System Biosciences, Palo Alto, CA, USA; Cat. No. EXFT20A-1). For 1 × MSC EV dose condition, 0.4 µL Exo-Fect reagent, 8 nmol cel-miR-39-3p mimic (Qiagen, Hilden, Germany; Cat. No. MSY0000010), and 10 µL transfection buffer were incubated at room temperature for 15 min. Subsequently, for 1 × condition, 6 × 10^8^ EVs suspended in 10 µL PBS was added to the transfection reaction and incubated at 37 °C for 1 h. The 2× and 4× conditions used proportionally increased inputs of 1.2 × 10^9^ particles with 16 nmol mimic and 2.4 × 10^9^ particles with 32 nmol mimic, respectively. PBS without EVs underwent the same Exo-Fect and ExoQuick processing and was used as no-EV process control. Other parallel control conditions included free cel-miR-39-3p mimic without EVs or Exo-Fect and unloaded MSC EVs mixed with and without cel-miR-39-3p mimic. EVs were re-isolated with ExoQuick (System Biosciences, Palo Alto, CA, USA; Cat. No. EXOQ5A-1). ExoQuick reagent was added to the transfection reaction at a 1:5 ratio (vol/vol) and incubated on ice for 30 min. The EVs were pelleted at 13,000× *g* for 3 min at 4 °C and resuspended in PBS and particle size distribution was analyzed with the NanoSight NS300 instrument and NTA software described above. RNase treatment was not included in the final Exo-Fect/ExoQuick protocol used to prepare the samples for the uptake experiments.

### 2.5. Relative Transfection Efficiency

Pre-transfection sample consisted of 1.5 µg H9C2 cellular RNA, 8 nmol cel-miR-39-3p mimic (Qiagen, Hilden, Germany; Cat. No. MSY0000010) and 6 × 10^8^ MSC EVs. Total RNA was isolated from the pre-transfection sample and the transfected EVs, and cDNA was synthesized using the TaqMan Advanced miRNA cDNA synthesis kit (Thermo Fisher Scientific, Waltham, MA, USA; Cat. No. A28007). Real-time PCR was performed to measure cel-miR-39-3p and normalized to miR-21a-5p, and the transfection efficiency was calculated by comparing the CT between pre-transfection and transfected EV samples by the ΔΔCT method [38]. Representative results are provided in Supplementary Figure S1. Because this was a relative CT-based measurement without an absolute standard curve, it does not provide the mass or copy number of cel-miR-39-3p associated with individual EVs, only recovery of cel-miR-39-3p in the re-isolated preparation. Because paired RNase treatment with and without membrane-disrupting detergent was not performed, the assay cannot distinguish intravesicular miRNA from miRNA associated with the EV surface or co-precipitated nonvesicular complexes.

### 2.6. Cytotoxicity Assay

H9C2 cells were subcultured in 96-well plates at a density of 1.5 × 10^4^ cells/well and allowed to attach and grow overnight. The next day, the cells were stained with Hoechst 33342 (Thermo Fisher Scientific, Waltham, MA, USA; Cat. No. H3570) at 0.2 µg/mL and propidium iodide (PI; Thermo Fisher Scientific, Waltham, MA, USA; Cat. No. P3566) at 2 µg/mL and imaged using a Cytation 5 imaging plate reader (BioTek Instruments, Winooski, VT, USA). After a wash with PBS, cells were treated with 5–50 µM chlorpromazine (CPZ; Sigma-Aldrich, St. Louis, MO, USA; Cat. No. 31679) for 2.5 h, or 10–100 µM 5-(N-Ethyl-N-isopropyl)-amiloride (EIPA; Sigma-Aldrich, St. Louis, MO, USA; Cat. No. A3085) for 2.5 h, or 10–50 µM ES9-17 (Sigma-Aldrich, St. Louis, MO, USA; Cat. No. SML2712) in DMEM for 2.5 h and stained again with Hoechst and PI. Dimethyl sulfoxide (DMSO; Sigma-Aldrich, St. Louis, MO, USA; Cat. No. D8418) was used as a vehicle control. Cell survival was calculated as the ratio of viable cell counts after treatment to viable cell counts before treatment, where viable cell counts were defined as the number of Hoechst-positive cells minus PI-positive cells, expressed as percentage.

### 2.7. Extracellular Vesicle Uptake Assay

H9C2 cells were subcultured in 12-well plates at a density of 1 × 10^5^ cells/well and allowed to attach and grow overnight. The next day, they were pretreated with 25 µM CPZ for 30 min, or 50 µM EIPA for 30 min, or 50 µM ES9-17 for 30 min, or 100 nM Antagomir-39-3p (5′-mC(*)mA(*)mAmGmCmUmGmAmUmUmUmAmCmAmCmCmCmGmG(*)mU(*)mG (*)mA(*)-3′-Chol) or Antagomir-scr (5′-mG(*)mC(*)mAmGmUmAmUmCmGmGmGmC mUmAmCmCmCmUmU(*)mA(*)mA(*)mA(*)-3′-Chol) for 2 h, then treated by adding cel-miR-39-3p-transfected EVs for 2 h at 6 × 10^8^ (1×), 1.2 × 10^9^ (2×) and 2.4 × 10^9^ (4×) particles/well. Both oligonucleotides were custom synthesized by Dharmacon, GE Healthcare (Lafayette, CO, USA). Cells were washed with PBS three times and total RNA was collected.

### 2.8. RNA Isolation

Total RNA was isolated from H9C2 cells. H9C2 cells were lysed and homogenized in QIAzol Lysis Reagent (Qiagen, Hilden, Germany; Cat. No. 79306), and total RNA was isolated by phenol/chloroform phase separation and precipitation in ice-cold isopropanol [39]. The pellet was washed with ice-cold 75% ethanol in RNAse-free water and resuspended in 80 µL of RNase/DNase-free water (Thermo Fisher Scientific, Waltham, MA, USA; Cat. No. 10977015) for analysis. Samples isolated for TaqMan Advanced Assay were aliquoted and thawed no more than once prior to their analysis in downstream assays.

### 2.9. Real-Time PCR

TaqMan Advanced miRNA cDNA synthesis kit (Thermo Fisher Scientific, Waltham, MA, USA; Cat. No. A28007) was used to reverse-transcribe the RNA for analysis of miRNA. Real-time PCR was performed on the AriaMx Real-Time PCR System (Agilent Technologies, Santa Clara, CA, USA) or QuantStudio 5 Real-Time PCR System (Applied Biosystems, Thermo Fisher Scientific, Waltham, MA, USA) in triplicate using TaqMan Fast Advanced Master Mix (Thermo Fisher Scientific, Waltham, MA, USA; Cat. No. 4444557), according to the manufacturer’s instructions. TaqMan Advanced miRNA Assays (Thermo Fisher Scientific, Waltham, MA, USA; Cat. No. A25576) were used for cel-miR-39-3p (Assay ID 478293_mir; mature sequence: 5′-UCACCGGGUGUAAAUCAGCUUG-3′) and miR-21a-5p (Assay ID mmu482709_mir; mature sequence: 5′-UAGCUUAUCAGACUGAUGUUGA-3′). The mature miR-21a-5p sequence targeted by this assay is identical in the mouse and rat. cel-miR-39-3p was normalized to endogenous miR-21a-5p and data were analyzed by the ΔΔCT method [38]. miR-21a-5p was selected as the endogenous reference because it is highly abundant in H9C2 cells and did not differ significantly among the experimental groups during the 2 h treatment period. The endogenous cellular miR-21a-5p pool greatly exceeded the amount associated with the MSC EV preparations; consequently, EV treatment did not measurably alter total cellular miR-21a-5p under these experimental conditions. It was used as the reference miRNA for the ΔΔCT calculations in both the relative transfection efficiency assessment and the recipient cell analyses. Its absolute abundance in the EV preparations was not determined.

### 2.10. Transferrin Endocytosis Assay

H9C2 cells were subcultured in 8-well chamber slides at a density of 4 × 10^4^ cells/well and allowed to attach and grow overnight. The next day, the cells were pretreated with 0.1% (*v*/*v*) DMSO vehicle or 25 µM CPZ for 30 min. Cells were cooled on ice for 10 min to suppress basal endocytosis before synchronized transferrin uptake at 37 °C. The cells were then washed with cold PBS and incubated for 15 min at 37 °C with Texas Red-conjugated transferrin (Thermo Fisher Scientific, Waltham, MA, USA; Cat. No. T2875) at 25 µg/mL and Hoechst 33342, as described above, at 1 µL/mL in the presence of the drug. The cells were washed and 4% PFA was used to fix the H9C2 cells on the slide for 10 min. Images were taken 24 h later with an Olympus IX81 inverted fluorescent microscope (Olympus Corporation, Tokyo, Japan) equipped with a Hamamatsu C11440-36U camera (Hamamatsu Photonics, Hamamatsu, Japan). Representative images were acquired using a 100× oil-immersion objective (NA 1.45), whereas images used for quantitative analysis were acquired using a 40× oil-immersion objective (NA 1.40). At least 40 images from at least four wells were analyzed per condition using ImageJ, version 1.54d (National Institutes of Health, Bethesda, MD, USA). For each condition, the fluorescence threshold was established from the mean intensity of at least five background regions. Cell areas were manually traced, and the background-subtracted integrated Texas Red fluorescence was divided by the number of cells in each image. Fluorescence intensity per cell was used to quantify transferrin uptake.

### 2.11. Experimental Design and Statistical Methods

Data are presented in superplot format [40]. Each color represents a different experiment repeat, small dots represent individual data points and large dots represent the mean of each repeat. Statistical analyses were conducted with GraphPad Prism, version 9.0 (GraphPad Software, San Diego, CA, USA). For normally distributed data with equal variance, comparisons between two conditions were made using Student’s *t*-test, and between 3 or more conditions using the ANOVA test. Group size was determined using a power analysis with a desired β of 0.8. Values were expressed as mean plus or minus standard error of the mean (SEM). Statistical significance between groups was set at *p* ≤ 0.05.

## 3. Results

### 3.1. MSC EV Deliver miRNA to Cardiomyocytes in a Dose-Dependent Manner

**Figure 1:**
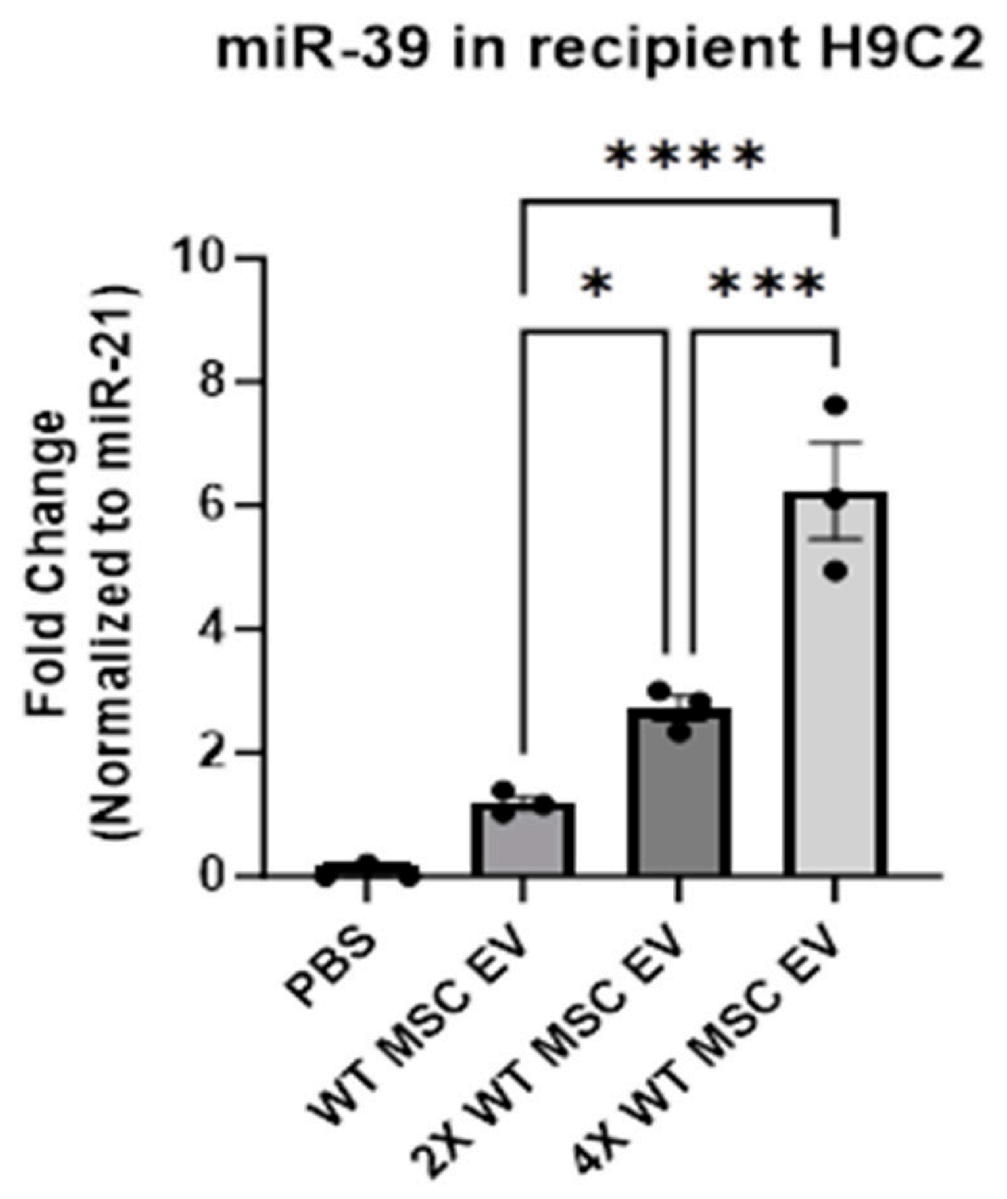
Transfected MSC EVs increases cel-miR-39-3p in cardiomyocytes in a dose-dependent manner. H9C2 cells were treated with 6 × 10^8^ (1×), 1.2 × 10^9^ (2×), and 2.4 × 10^9^ (4×) MSC EVs transfected with 8 nmol (1×), 16 nmol (2×), and 32 nmol (4×) cel-miR-39-3p for 2 h. The stated miRNA quantities represent loading inputs and not measured post-isolation EV-associated amounts. The no-EV process control (PBS) contained cel-miR-39-3p, Exo-Fect reagent, and transfection buffer but contained PBS in place of EVs and underwent the same ExoQuick re-isolation procedure. Cellular RNA was collected, and qRT-PCR analysis was performed. cel-miR-39-3p levels were normalized to miR-21a-5p levels. * *p* ≤ 0.05, *** *p* ≤ 0.001, **** *p* ≤ 0.0001 by one-way ANOVA with Fisher’s LSD test. Data bars represent mean ± SEM. Abbreviations: ANOVA, analysis of variance; EV, extracellular vesicle; LSD, least significant difference; miRNA, microRNA; MSC, mesenchymal stem cell; PBS, phosphate-buffered saline; qRT-PCR, quantitative reverse transcription polymerase chain reaction; SEM, standard error of the mean.

To determine whether MSC EVs can deliver miRNAs into cardiomyocytes, we first sought to establish a model that avoided possible EV induction of endogenous miRNAs as a limitation to measurement, so we loaded EVs with cel-miR-39-3p mimic as exogenous miRNA cargo. The Exo-Fect/ExoQuick procedure produced a mean relative transfection efficiency of 69.9 ± 21.9% (n = 3) compared with the pre-transfection comparator (Supplementary Figure S1). This CT-based value represents relative recovery of cel-miR-39-3p in the re-isolated preparation and not an absolute miRNA amount or the fraction encapsulated within EVs. Following increasing doses of cel-miR-39-transfected MSC EVs, H9C2 cardiomyocytes showed increased cel-miR-39-3p levels in a dose-dependent manner (Figure 1). The identically processed no-EV control (PBS) also produced a detectable cel-miR-39-3p signal, although the signal was lower than that produced by the EV-containing preparations. Exo-Fect/ExoQuick processing also generated a detectable nanoparticle population in the no-EV control, with a representative concentration of 2.29 × 10^9^ particles/mL compared with 3.42 × 10^10^ particles/mL in the EV-containing preparation (Supplementary Figure S2). Free cel-miR-39-3p mimic, unloaded MSC EVs, and MSC EV mixed with cel-miR-39-3p mimic were also evaluated as controls. None of these conditions produced detectable cel-miR-39-3p signal in recipient-cell RNA under the assay conditions. Because the final Exo-Fect/ExoQuick preparations were not treated with RNase, a contribution from externally accessible or nonvesicular Exo-Fect-associated miRNA cannot be excluded. [41]. Here, “×” denotes fold scaling of the loading-reaction inputs relative to the 1× condition; it does not denote the measured amount of miRNA in the final preparation. The 1×, 2×, and 4× reactions contained 8, 16, and 32 nmol cel-miR-39-3p mimic and 6 × 10^8^, 1.2 × 10^9^, and 2.4 × 10^9^ starting EV particles, respectively. The absolute amount of cel-miR-39-3p associated with the final re-isolated EV-enriched preparation was not determined. Endogenous miR-21a-5p content of the EV preparation was also not measured because miR-21a-5p was used only to normalize cel-miR-39-3p measured in recipient-cell RNA.

### 3.2. MSC EV Can Deliver miRNA Cargo into Cardiomyocytes

**Figure 2:**
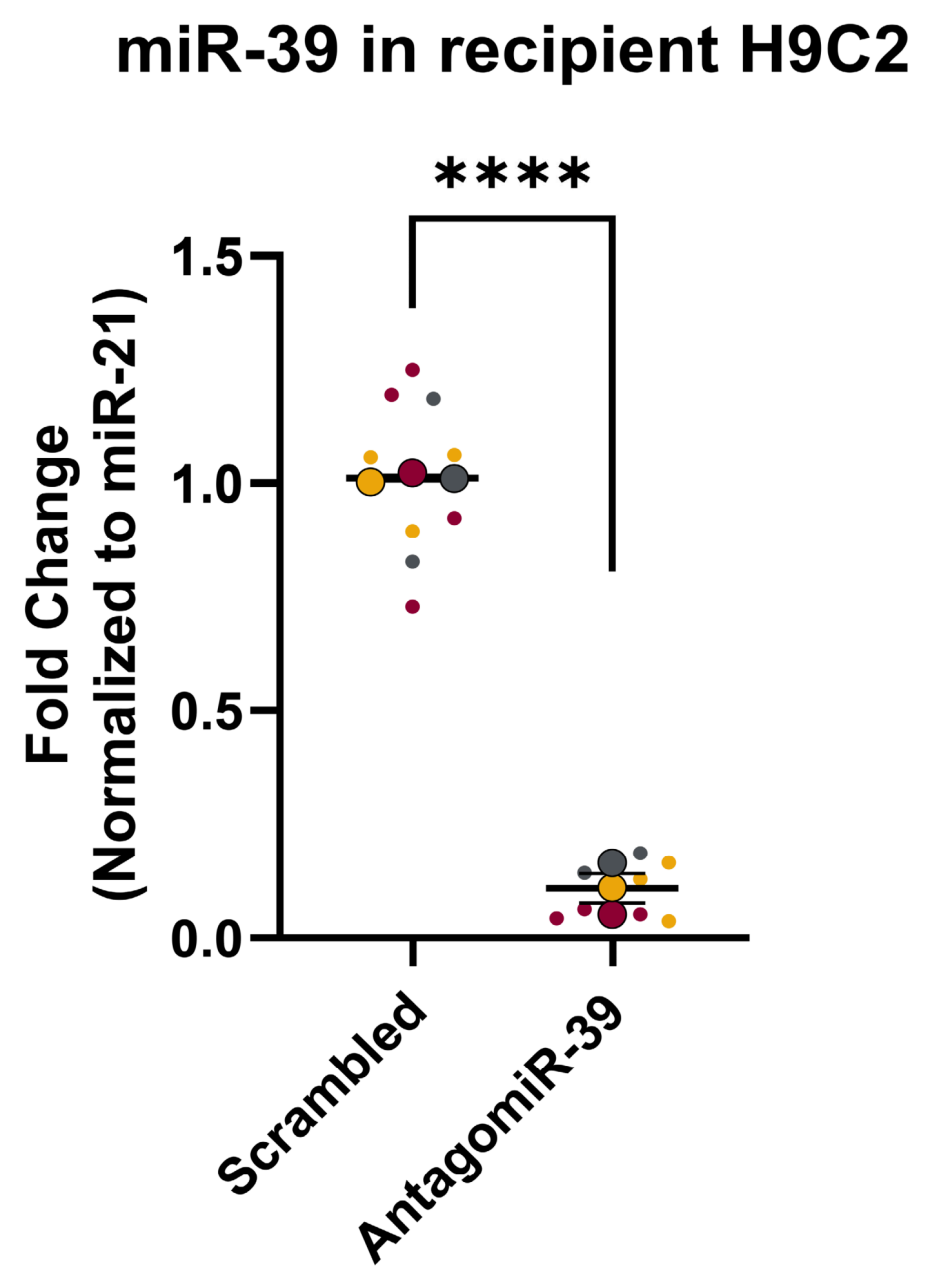
Antagomir-39-3p reduces cell-associated cel-miR-39-3p in H9C2 cells. H9C2 cells were pretreated with 100 nM sequence-scrambled oligo or antagomir-39 oligo in serum-free media for 2 h. Cells were washed and treated with cel-miR-39-transfected MSC EVs for 2 h (only 2× EV dose shown). Cellular RNA was collected, and qRT-PCR analysis was performed. cel-miR-39-3p levels were normalized to miR-21a-5p levels. Data are presented as a superplot. Three colors represent three different experimental repeats, small dots represent individual data points, and large dots represent the mean of each repeat. **** *p* ≤ 0.0001 by unpaired *t*-test. Data bars represent mean ± SEM. Abbreviations: EV, extracellular vesicle; miRNA, microRNA; MSC, mesenchymal stem cell; qRT-PCR, quantitative reverse transcription polymerase chain reaction; SEM, standard error of the mean.

To determine whether miRNAs from MSC EVs enter cardiomyocytes or only adhere to the cell surface, we designed an antagomir inhibitor of cel-miR-39-3p with previously established modifications [33]. The 2′-O-methyl modification and phosphorothioate modification protect the oligo from hydrolysis and RNase activity, and the cholesterol modification on the 3′-end assists oligo cell entry without exogenous transfection agent. Following 2 h incubation with the oligos in serum-free media, the H9C2 cells were washed and treated with cel-miR-39-transfected MSC EVs, as described above. Antagomir-39-3p significantly reduced the cel-miR-39-3p levels detected in H9C2 cells compared to the chemically matched sequence-scrambled control (Figure 2), demonstrating high inhibition efficiency under the experimental conditions. The matched scrambled control supports sequence-dependent activity of antagomir-39-3p, although it does not exclude all possible off-target or cytotoxic effects. Taken together, the results provide indirect functional evidence that a substantial portion of the MSC EV-derived cel-miR-39-3p reached an antagomir-accessible cytosolic compartment of H9C2 cardiomyocytes. However, because cel-miR-39-3p was not directly visualized with any compartment marker, its distribution between endosomal and cytosolic compartments was not quantified.

### 3.3. EIPA Reduces MSC-Associated Cel-miR-39-3p in Cardiomyocytes

**Figure 3:**
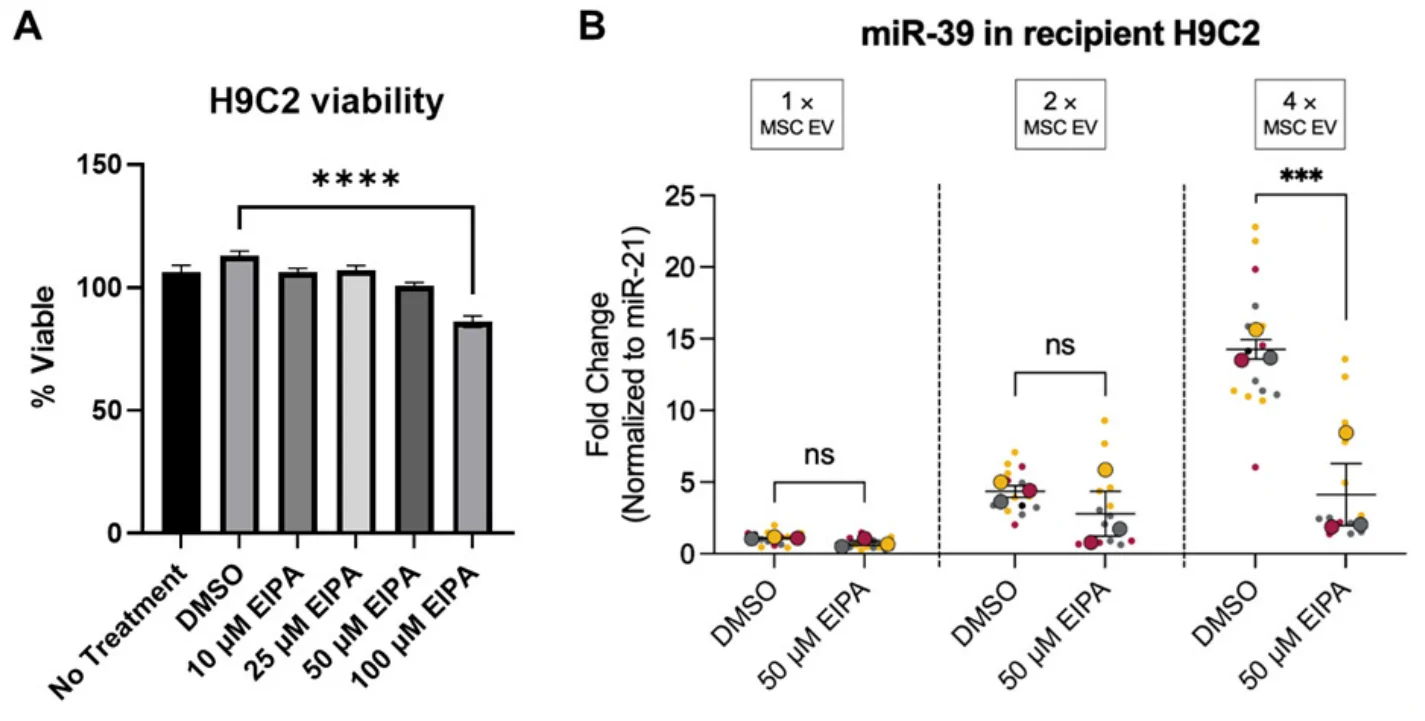
EIPA reduced MSC EV miRNA delivery into cardiomyocytes. (**A**) Percentage of H9C2 survival after 2.5 h treatment. **** *p* ≤ 0.0001 by one-way ANOVA with Tukey’s correction for multiple comparisons. Data bars represent mean ± SEM. (**B**) H9C2 cells were pretreated with 50 µM EIPA for 30 min and treated with MSC EVs transfected with cel-miR-39-3p for 2 h as described. Cellular RNA was collected, and qRT-PCR analysis was performed. cel-miR-39-3p levels were normalized to miR-21a-5p levels. Data are presented as a superplot. Three colors represent three different experimental repeats, small dots represent individual data points, and large dots represent the mean of each repeat. *** *p* ≤ 0.001 by two-way ANOVA with Tukey’s correction for multiple comparisons. Data bars represent mean ± SEM. Abbreviations: ANOVA, analysis of variance; EIPA, 5-(N-ethyl-N-isopropyl) amiloride; EV, extracellular vesicle; miRNA, microRNA; MSC, mesenchymal stem cell; qRT-PCR, quantitative reverse transcription polymerase chain reaction; SEM, standard error of the mean.

Next, we investigated the mechanisms of MSC EV endocytosis into cardiomyocytes. We first examined whether macropinocytosis is involved in this process, using EIPA as a pharmacological inhibitor. EIPA is commonly used to inhibit macropinocytosis by disrupting Na^+^/H^+^ exchange-dependent signaling [42,43]. Consistent with published studies, the maximal non-toxic dose of EIPA for H9C2 cells following a 2.5 h treatment was 50 µM (Figure 3A). We then pretreated the H9C2 cells with 50 µM EIPA for 30 min and added three doses of cel-miR-39-transfected MSC EVs for 2 h, as above. The qRT-PCR of the cells showed that 50 µM EIPA significantly reduced cel-miR-39-3p levels associated with H9C2 cells following the highest MSC EV dose treatment compared to DMSO vehicle control (Figure 3B). These results suggest an EIPA-sensitive uptake process consistent with macropinocytosis.

### 3.4. Pharmacologic Perturbation of Clathrin-Associated Uptake Alters Cel-miR-39-3p Accumulation

**Figure 4:**
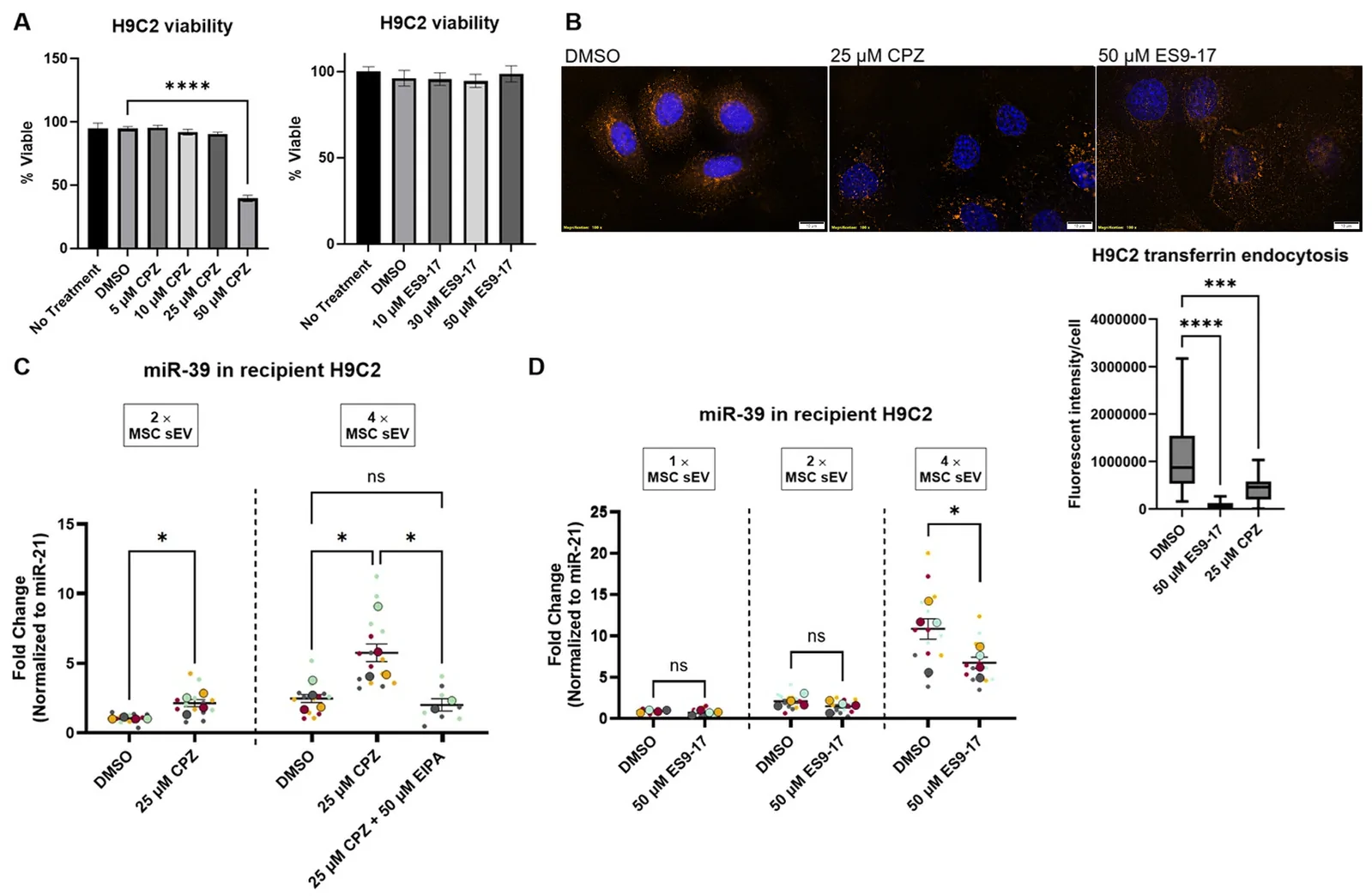
ES9-17 but not CPZ inhibited MSC EV miRNA delivery into cardiomyocytes. (**A**) Percentage of H9C2 survival after 2.5 h treatment. **** *p* ≤ 0.0001 by one-way ANOVA with Tukey’s correction for multiple comparisons. Data bars represent mean ± SEM. (**B**) Representative images (top) of H9C2 cells treated with Texas Red conjugated transferrin following pretreatment with DMSO or 25 µM CPZ or 50 µM ES9-17 for 30 min. Scale bars = 10 µm. Quantification of fluorescent intensity/cell (bottom) analyzed with ImageJ. The number of cells analyzed for DMSO, CPZ, and ES9-17 groups is 1578, 2055, and 1035, respectively (n = 3). *** *p* ≤ 0.001 DMSO vs. CPZ, **** *p* ≤ 0.0001 DMSO vs. ES9-17 by Kruskal–Wallis test with Dunn’s correction for multiple comparisons. Data bars represent min to max. (**C**) H9C2 cells were pretreated with 25 µM CPZ for 30 min and treated with MSC EVs transfected with cel-miR-39-3p for 2 h as described. Cellular RNA was collected, and qRT-PCR analysis was performed. cel-miR-39-3p levels were normalized to miR-21a-5p levels. Data are presented as a superplot. Four colors represent different experimental repeats, small dots represent individual data points, and large dots represent the mean of each repeat. * *p* ≤ 0.05 by two-way ANOVA with Tukey’s correction for multiple comparisons. Data bars represent mean ± SEM. (**D**) H9C2 cells were pretreated with 50 µM ES9-17 for 30 min and treated with MSC EVs transfected with cel-miR-39-3p for 2 h as described. Cellular RNA was collected, and qRT-PCR analysis was performed. cel-miR-39-3p levels were normalized to miR-21a-5p levels. Data are presented as a superplot. Four colors represent different experimental repeats, small dots represent individual data points, and large dots represent the mean of each repeat. * *p* ≤ 0.05 by two-way ANOVA with Tukey’s correction for multiple comparisons. Data bars represent mean ± SEM. Abbreviations: ANOVA, analysis of variance; CPZ, chlorpromazine; DMSO, dimethyl sulfoxide; ES9-17, clathrin heavy-chain inhibitor; EV, extracellular vesicle; miRNA, microRNA; MSC, mesenchymal stem cell; qRT-PCR, quantitative reverse transcription polymerase chain reaction; SEM, standard error of the mean.

Given multiple reports on MSC EV entering different cells through clathrin-mediated endocytosis, we decided to examine if that is the case for cardiomyocytes. We first used a well-known clathrin-mediated endocytosis inhibitor, chlorpromazine (CPZ). We established that the non-toxic dose of CPZ for H9C2 with 2.5 h treatment time is 25 µM (Figure 4A). We then pretreated the H9C2 cells with 25 µM CPZ for 30 min and added cel-miR-39-transfected MSC EVs for 2 h, as above. Interestingly, the qRT-PCR of the cells showed that 25 µM CPZ not only did not reduce cel-miR-39-3p levels associated with H9C2 cells, but significantly increased cel-miR-39-3p levels in a dose-dependent manner compared to DMSO vehicle control. The addition of 50 µM EIPA reduced cel-miR-39-3p to levels comparable to the DMSO control (Figure 4C). We confirmed that 25 µM CPZ inhibited clathrin-mediated endocytosis of transferrin (Figure 4B), making the induction of cel-miR-39-3p by CPZ even more anomalous.

Because CPZ increased rather than decreased cel-miR-39-3p accumulation despite inhibiting transferrin uptake, we then used a potent and selective inhibitor of clathrin heavy chain, ES9-17 to inhibit clathrin-mediated endocytosis. We found that ES9-17 did not affect H9C2 cell viability at high doses for the duration of our experiment (Figure 4A) and at 50 µM dose, it inhibited clathrin-mediated endocytosis of transferrin into H9C2 cells (Figure 4B). When we pretreated H9C2 cells with 50 µM ES9-17 for 30 min and added cel-miR-39-transfected MSC EVs for 2 h, the qRT-PCR results showed that 50 µM ES9-17 reduced cel-miR-39-3p levels associated with H9C2 cells following the highest MSC EV dose treatment (Figure 4D). The reduction produced by ES9-17, together with its inhibition of transferrin uptake, implicates a clathrin-associated process. However, the paradoxical increase produced by CPZ and the absence of genetic perturbation preclude definitive assignment of clathrin-mediated endocytosis as the causal uptake pathway.

## 4. Discussion

Since their discovery, extracellular vesicles (EVs) have been gaining increasing attention for their role as gene regulatory paracrine factors, and their therapeutic potential, including the novel route of drug administration and potential cell-type-specific targeting. In the heart, the crosstalk between cardiomyocytes and myofibroblasts can affect fibroblast proliferation, cardiac hypertrophy, and fibrosis [44,45]. The paracrine hypothesis asserts that stem cells release factors, including EVs that affect adaptation to conditions in the myocardium [46,47]. It was thought that the uptake of stem cell EVs by the cardiac cells mediates the transfer of cardioprotective cargo. Previously, we showed that the addition of bone marrow-derived MSC EVs leads to a downregulation of apoptotic pathways through microRNA-mediated signaling in mouse models. Specifically, the increase in miR-21a-5p following MSC EV treatment was shown to facilitate this effect [10]. This work expands the understanding of the MSC EV miRNA transfer in the heart and provides a new approach for studying RNA cargo transfer between EVs and cells. Furthermore, understanding EV endocytosis is advantageous in that the specific mechanisms can be manipulated for therapeutic purposes, such that induction of major endocytic pathways can lead to better therapeutic efficacy. In the cancer field where EVs are better studied, macropinocytosis has been targeted for therapeutic development [48]. In aggressive and metastatic cancers that utilize macropinocytosis for nutrient uptake in hypoxia and nutrient-deprived environments, inhibition of this process can reduce metastatic potential [49,50,51,52]. On the other hand, excessive fluid uptake via macropinocytosis is recognized as a process of methuosis, which can create large vacuoles and cell swelling that lead to nonapoptotic cell death [53,54].

The goal of this study was to understand how EVs deliver cardioprotective miRNAs in the heart. The use of cel-miR-39-3p as EV cargo solved the issue of potential background or endogenous expression of the same miRNA. Many previous studies have focused on functions of EV contents in the heart. Rarely has there been a rigorous determination of whether miRNA contents are directly transferred into the recipient cells. In fact, many such studies are consistent with delivery of miRNAs or induction of endogenous miRNA genes with EV binding. The endocytic process relies on the different surface proteins of the EVs and the cells, and no study has rigorously shown direct evidence of MSC EV miRNA transfer into cardiomyocytes. A few studies have used knockout recipient cells or exogenous miRNAs to show transfer of EV cargo. Ong et al. transfected mouse endothelial cells with cel-miR-39 and collected EVs from them, but it is unclear whether the treatment had any effects on the EV miRNA sorting mechanism in the cells [18]. cel-miR-54 is another suitable non-mammalian miRNA and has previously been used to examine EV-mediated miRNA transfer in mouse and human recipient cells [19]. More generally, candidate miRNAs should be absent from the donor and recipient genomes, have negligible baseline signals and a validated detection assay, and be screened for unintended mammalian targets or immunostimulatory effects.

The hypothesis of this study was that MSC EVs transfer functional miRNA cargo into cardiomyocytes through endocytosis. The transfer of EV miRNA cargo was confirmed in that the antagomir-39-3p treatment resulted in an 89% decrease in cel-miR-39-3p levels in the cardiomyocyte following transfected MSC EV treatment (Figure 2). Krützfeldt et al. established in a different cell system that these modified antagomirs are located in the cytosol and interact with their target miRNA in a compartment upstream of P-bodies, leading to miRNA degradation [33]. These findings provide indirect evidence that MSC EV-delivered cel-miR-39-3p reaches a functional cytoplasmic compartment within recipient cardiomyocytes, where it is accessible to antagomir-mediated degradation. However, following endocytic uptake, cel-miR-39-3p may initially remain within early endosomes, progress to late endosomes or lysosomes, recycle to the extracellular space, or escape into the cytosol. Neither cel-miR-39-3p nor antagomiR-39 was directly localized in H9C2 cells. The experiment therefore does not quantify the proportion of cel-miR-39-3p retained within early or late endosomes relative to the proportion reaching the cytosol. Although a chemically matched sequence-scrambled oligonucleotide was included, antagomir-specific effects on H9C2 viability and broader off-target effects were not independently assessed. Notably, the cytoplasm is the cellular compartment in which miRNAs engage their mRNA targets and exert canonical regulatory activity. Unlike studies that infer transfer solely from increased microRNA abundance in recipient cells, the antagomir accessibility assay provides evidence that EV-delivered microRNA reaches a functional cytoplasmic compartment capable of supporting canonical microRNA regulatory interactions.

The transfected MSC EVs demonstrated a dose-dependent increase in cel-miR-39-3p transfer into cardiomyocytes. Results from inhibitor studies indicate that EIPA and ES9-17 decreased the cel-miR-39-3p levels detected in the recipient cardiomyocytes at the highest preparation dose by 71% and 36%, respectively (Figure 3B and Figure 4D), implicating EIPA-sensitive and clathrin-associated uptake processes. However, these pharmacologic results do not establish causal pathway dependence because the inhibitors may have off-target effects and the relevant pathway components were not genetically perturbed. These results also indirectly support the transfer of EV miRNA into the cytoplasm from EVs. This consideration came up in pondering the rigor of our work. EVs could stick or be bound to the cell surface and have their contents isolated along with cellular RNA. The miRNAs adherent to the cell surface could be detected in the qRT-PCR, a consideration that is rarely made in the field. Because RNase treatment was not included in the final Exo-Fect/ExoQuick protocol, the qRT-PCR measurements cannot fully exclude externally accessible or nonvesicular Exo-Fect-associated miRNA. However, the fact that antagomirs work and that endocytic inhibitors reduced the levels so significantly argues that we are truly measuring EV cargo that has been taken into the cells. While the 4× MSC EV treatment gave a signal and showed a significant reduction with ES9-17, we noted that the 1× and 2× MSC EV treatments did not show a significant difference relative to DMSO controls (Figure 4D). This is likely due to the low dosage of EVs used and the short treatment time. Our laboratory previously demonstrated uptake of mouse MSC EVs by H9C2 cells using PKH26 flow cytometry and ImageStream analysis, as well as covalent maleimide-Alexa Fluor 488 labeling [10]. More than 90% of H9C2 cells were PKH26-positive after overnight exposure, and covalently labeled EV signal was detected intracellularly after 4 h. Together with the absence of endogenous cel-miR-39-3p and the lack of detectable recipient cell signal from free cel-miR-39-3p or unloaded EVs, these findings support the use of recipient cell cel-miR-39-3p as a specific surrogate for MSC EV uptake-mediated cargo transfer in the present study.

One interesting result from the study is that CPZ, a widely accepted clathrin-mediated endocytosis inhibitor, showed a significant increase in MSC EV cel-miR-39-3p transfer into H9C2 cells (Figure 4C), while the same dose of 25 µM was enough to inhibit transferrin endocytosis (Figure 4B). One report from Demasi et al. showed that CPZ can enhance iron uptake from citrate and transferrin donors to rat brain cortical synaptosomes [55]. They also showed the interplay between calcium and CPZ in iron and calcium uptake by the synaptosomes, which could lead to the development of neurological disorders. However, this study used a higher dose of CPZ at 50-250 µM, which could cause not only higher toxicity, but also off-target effects from nonspecific binding of CPZ. A possible mechanism is that CPZ changes the cell membrane curvature, which is known to affect the endocytic process. CPZ is a cationic molecule and would preferentially insert into the more negatively charged inner leaf of the plasma membrane, causing curvature, which could aid in the endocytic vesicle formation and increased cellular endocytosis [56,57,58]. The reversal of the CPZ-associated increase in cel-miR-39-3p by EIPA is suggestive of a contribution of macropinocytosis to the CPZ response, although the data cannot distinguish compensatory pathway activation from an EIPA-sensitive off-target effect. (Figure 4C). On the contrary, studies have shown that 10 µM CPZ can block the cardioprotective effects of adipose-derived regenerative cells in animal MI and cell-conditioned medium models in vitro [32]. CPZ can also inhibit the uptake of normal and pre-eclamptic syncytiotrophoblast-derived EVs by endothelial cells [59]. Taken together with our other inhibitor experiments, we speculate that CPZ increases EIPA-sensitive or other endocytic pathways in cardiomyocytes by altering cell membrane curvature. However, the present data cannot distinguish compensatory activation of an alternative pathway from sequential or cooperative uptake, altered membrane curvature, or another off-target effect of CPZ, and further studies are needed to determine the exact mechanism of CPZ effects on cardiomyocyte endocytosis.

In our study, we demonstrate recipient cell cel-miR-39-3p was accessible to a cytosolic antagomir, providing functional evidence that miRNA associated with MSC EVs reached an antagomir-accessible cytoplasmic compartment in H9C2 cardiomyocytes. The effects of EIPA and ES9-17 implicate EIPA-sensitive uptake consistent with macropinocytosis and clathrin-associated uptake, respectively. Together with our previous studies demonstrating MSC EV uptake and the cardioprotective effects of MSC EV-associated miRNAs, the present findings provide mechanistic support for transfer of MSC EV microRNA cargo into cardiomyocytes where they elicit cardioprotective effects by inhibiting gene expression of cell death proteins, including PTEN, PDCD4 and FasL [10]. In addition to miRNAs, many studies have demonstrated that EVs can transfer functional mRNA, lncRNA, and DNA plasmid into cells and thereby regulate gene expression in the recipient cells [60,61,62,63]. These results contribute to the idea that different EV populations utilize different mechanisms for cargo transfer. By understanding how MSC EVs deliver their cargo into cardiac cells, we can manipulate this process for the development of cell-free molecular therapeutic approaches for cardiovascular diseases.

A methodological contribution of the present study is the combined use of a non-mammalian miRNA tracer with negligible recipient-cell background, proportional loading inputs, and a sequence-matched antagomir control. This design complements commonly used EV-uptake approaches. Chief among them is visualization of EV uptake with lipophilic fluorescent dyes, such as PKH dyes and DiO/DiI dyes. Though few studies rely on confocal microscopy alone, it is not recommended to use lipophilic dyes to label EVs as these dyes do not covalently label, tend to self-aggregate and can generate high noise and false signals. Use of covalent dye-labeled miRNAs, including maleimide dye, is much more reliable [64,65]. Another widely used design for demonstrating miRNA transfer is the overexpression of the miRNA in the EV-producing cells or the EVs themselves [66]. These methods could affect the natural production or content of EVs. A derivative of this method is simply demonstrating an increase in miRNA in the recipient cells when treated with miRNA containing EVs [67]. Some studies, on the other hand, employ loss-of-function design to deplete EV-producing cells of the miRNA of interest or using antagomirs as EV cargo [68,69,70]. However, the knockdown of the miRNAs of interest in the EV-producing cells does not provide the same strength in evidence compared with KO cell models. These manipulations of EV-producing cells and their EVs share the disadvantage that, without a clean background in the recipient cells, the changes in miRNA in the recipient cells can be attributed to other possible EV factors or signaling that led to changes in endogenous miRNA expression levels, rather than direct miRNA transfer. Compared to the generation of KO lines, the method in this study is easy to perform and can be used to study EV endocytosis in most cell types. The demonstration of dose effects (Figure 1) and the use of antagomir controls (Figure 2) provide easy determination of transfer and function. Therefore, we encourage future studies to employ similar experiments to demonstrate EV cargo transfer with scientific rigor.

Recent methodological advances also emphasize the importance of EV preservation and subpopulation-specific analysis. Geng et al. identified PBS supplemented with 25 mM trehalose as an effective buffer for maintaining EV stability during short-term storage at −80 °C [71], whereas Liu et al. developed an antibody-assisted acoustofluidic platform for rapid, surface-marker-specific capture and detection of small EVs from low-volume samples [72]. Such approaches may improve the preservation, reproducibility, and characterization of EV preparations in future cargo-transfer studies.

## 5. Limitations

One major limitation of the study is the lack of evaluation of a potential biological function of cel-miR-39-3p in cardiac cells. The antagomir experiment provides indirect functional evidence that transferred MSC EV cel-miR-39-3p reached an antagomir-accessible cytosolic compartment, but cel-miR-39-3p was not directly localized in H9C2 cells. Direct confirmation would require a complementary approach, such as cel-miR-39-3p RNA-FISH with EEA1 and other compartment markers or biochemical subcellular fractionation with validated cytosolic and endosomal purity controls. Consequently, the study cannot quantify cel-miR-39-positive endosomes or determine the relative proportions of miRNA retained within endosomal compartments and released into the cytosol. In addition, membrane labeling was not repeated under the present 2 h inhibitor conditions; therefore, the cel-miR-39-3p assay does not directly quantify intact EV uptake in each treatment group. However, because there are no endogenous targets of cel-miR-39-3p in mammalian cells and no commercially available luciferase reporter for it, we could not study the function of cel-miR-39-3p in cardiomyocytes. Previously [10], we showed that the addition of MSC EVs leads to a downregulation of apoptotic pathways through an increase in miR-21a-5p and its mediated signaling in a mouse model. Though the transfer of cel-miR-39-3p is not equivalent to the transfer of miR-21a-5p, taken together, we can conclude that MSC EVs deliver miRNAs into cardiomyocytes to elicit cardioprotective effects.

Another limitation of this study is the use of the Exo-Fect reagent. While electroporating EVs can change their morphology and size to over 2 µm, transfecting EVs with Exo-Fect does not significantly alter their size (Supplementary Figure S2). However, results from the no-EV (PBS) controls indicate that the Exo-Fect reagent can form particles with similar shape and size as EVs, though to a small extent. These particles can even transfect cells and produce detectable levels of cel-miR-39-3p (Figure 1, first bar). In contrast, free cel-miR-39-3p mimic and MSC EVs mixed with or without cel-miR-39-3p produced no detectable cel-miR-39-3p signal in recipient cells, indicating that the background was associated with the Exo-Fect/ExoQuick procedure rather than spontaneous uptake of free mimic or endogenous expression. The final Exo-Fect/ExoQuick preparations were not treated with RNase. During preliminary assay development, RNase reduced cel-miR-39-3p levels in both PBS and EV-containing Exo-Fect preparations but did not eliminate the PBS control signal, suggesting the presence of both externally accessible miRNA and RNase-resistant non-EV material. Consequently, the CT-based transfection efficiency analysis reflects relative recovery but does not quantify the absolute amounts of cel-miR-39-3p or miR-21a-5p or the fraction of cel-miR-39-3p encapsulated within EVs. The present results therefore support transfer of cel-miR-39-3p associated with the EV preparation but do not definitively establish transfer of exclusively intravesicular miRNA.

The present experiments assessed recipient cell cel-miR-39-3p at a single 2 h endpoint and therefore do not define the kinetics of EV uptake, cytoplasmic miRNA appearance, intracellular persistence, or degradation. The antagomir experiment supports accessibility of cel-miR-39-3p within a functional cytoplasmic compartment at the 2 h endpoint, but determining the timing and persistence of cargo delivery will require future time-course and pulse-chase studies.

The 50 µM EIPA concentration was selected as the highest tested concentration that preserved H9C2 cell viability; however, it was submaximal within the tested concentration range and below the 150 µM concentration used with a fluorescent-dextran target-engagement assay in another cell system [73]. Incomplete pathway inhibition may therefore have caused the contribution of macropinocytosis to be underestimated. Because EIPA-mediated inhibition was not independently verified in H9C2 cells using a fluid-phase uptake assay such as fluorescent dextran, the degree of pathway blockade remains uncertain [73,74]. The reduction in cel-miR-39-3p is therefore interpreted as evidence of an EIPA-sensitive process consistent with macropinocytosis rather than definitive pathway-specific inhibition.

Because H9C2 cells are an immortalized rat cardiomyoblast-derived cell line used with mouse MSC EVs, species-specific differences in EV-cell interactions cannot be excluded; future studies should validate these findings in species-matched primary cardiomyocytes or human iPSC-derived cardiomyocytes.

More generally, pathway assignment in this study was based on pharmacologic perturbation. Although the selected inhibitor concentrations preserved cell viability and the effects of CPZ and ES9-17 on transferrin uptake were assessed, off-target effects cannot be excluded. Genetic knockdown or knockout of *Rac1* or *Cdc42* and clathrin-associated components such as *Cltc*, *Ap2m1*, or *Dnm2*, ideally accompanied by rescue experiments, would be required to establish causal pathway dependence. The present findings are therefore interpreted as evidence for EIPA-sensitive and clathrin-associated uptake processes rather than definitive proof of macropinocytosis or clathrin-mediated endocytosis. Though other mechanisms such as caveolin-mediated endocytosis and lipid rafts lack specific pharmacological antagonists, future studies using a combination of drugs or KO models may shed light on whether those mechanisms are involved in MSC EV endocytosis into cardiomyocytes. In addition, a complete factorial comparison of EIPA and ES9-17, individually and in combination, was not performed. Therefore, time-resolved experiments with validated individual and combined pathway perturbations are needed to determine how EIPA-sensitive and clathrin-associated processes operate together.

## Figures and Tables

**Table 1:** Selected evidence for EV miRNA transfer into recipient cells.

Method	Citation	EVs	Recipient Cells
KO recipient cell	Momen-Heravi et al., 2014 [11]	miR-155 mimic-electroporated murine B cell-derived EV	miR-155 KO primary mouse hepatocytes
	Ying et al., 2017 [12]	Mouse adipose tissue macrophage-derived EV	miR-155 KO mouse hepatocytes
	Nelson et al., 2024 [13]	cetuximab-resistant colorectal cancer cell-derived EV	miR-100/miR-125b KO colorectal cancer cells
Labeling	Valadi et al., 2007 [4] *	[^3^H]-uracil-labeled mouse mast cell-derived EV	Mouse and human mast cells
	Feng et al., 2014 [14] *^#^	Fluorescein-labeled miR-22 transfected mouse MSC co-culture	Murine cardiomyocytes
	Fu et al., 2023 [15]	Human umbilical cord MSC-derived EVs loaded by sonication with graphene quantum dot-bound Cy5-labeled miR-193a-3p	HGC-27 human gastric cancer cells and an in vivo xenograft model
Exogenous miRNA	Pegtel et al., 2010 [16]	Epstein–Barr virus-infected human lymphoblastoid B cell co-culture	Human monocyte-derived dendritic cells
	Buck et al., 2014 [17]	*H. polygyrus*-derived EV	Mouse small intestinal epithelial cells
	Ong et al., 2014 [18] *^#^	cel-miR-39-transfected mouse endothelial cell-derived EV	Mouse cardiac progenitor cells
	Nguyen et al., 2018 [19] *^#^	cel-miR-54-transfected mouse and human macrophage co-culture	Mouse and human naïve macrophages
	He et al., 2023 [20]	*Dermatophagoides farinae*-derived EV	Human bronchial epithelial BEAS-2B cells
Endogenous miRNA production removal	Okoye et al., 2014 [21]	Mouse Treg cell co-culture	*Dicer1* KO or *Rab27a/Rab27b* KO mouse conventional T cells

“Method” refers to the experimental manipulation used to ensure that material detected comes from outside the recipient cell. “EVs” refers to the EV producer cells used for the work. “Recipient cells” refers to the cell that was exposed to EVs. All studies except Buck et al. were done in vitro. * Lacks demonstration that EV-RNA had functional effect in recipient cells. ^#^ Representative example; studies with similar methods are not listed here. Abbreviations: ^3^H, tritium; Cy5, cyanine 5; EV, extracellular vesicle; KO, knockout; miRNA, microRNA; MSC, mesenchymal stem cell; Treg, regulatory T cell.

## Data Availability

The datasets generated and analyzed during the current study are available from the corresponding author upon reasonable request.

## Supplementary Materials

The following supporting information can be downloaded at https://www.mdpi.com/article/10.3390/biomedicines14092002/s1, Figure S1: Relative recovery of cel-miR-39-3p after Exo-Fect/ExoQuick processing; Figure S2: Exo-Fect and ExoQuick treatment does not significantly alter MSC EV size distribution; Figure S3: PBS control forms nanoparticles following Exo-Fect treatment.

## Abbreviations

The following abbreviations are used in this manuscript:

ANOVA	Analysis of variance
cDNA	Complementary DNA
CD	Cluster of differentiation
CPZ	Chlorpromazine
CT	Cycle threshold
Cy3	Cyanine 3
ΔΔCT	Delta–delta cycle threshold
DMEM	Dulbecco’s Modified Eagle Medium
DMSO	Dimethyl sulfoxide
DNA	Deoxyribonucleic acid
EIPA	5-(N-ethyl-N-isopropyl) amiloride
EV(s)	Extracellular vesicle(s)
FasL	Fas ligand
FBS	Fetal bovine serum
IL	Interleukin
IMDM	Iscove’s Modified Dulbecco’s Medium
KO	Knockout
lncRNA	Long non-coding RNA
LSD	Least significant difference
MI	Myocardial infarction
miR/miRNA	microRNA
MISEV2023	Minimal Information for Studies of Extracellular Vesicles 2023
mRNA	Messenger RNA
MSC(s)	Mesenchymal stem cell(s)
PBS	Phosphate-buffered saline
PDCD4	Programmed cell death 4
PFA	Paraformaldehyde
PI	Propidium iodide
PTEN	Phosphatase and tensin homolog
qRT-PCR	Quantitative reverse transcription polymerase chain reaction
RNA	Ribonucleic acid
RNase/DNase	Ribonuclease/Deoxyribonuclease
Sca1	Stem cell antigen-1
scr	Scrambled
SEM	Standard error of the mean
TGF-β1	Transforming growth factor beta 1
TSG101	Tumor susceptibility gene 101

## References

[1] Borges F.T., Reis L.A., Schor N. (2013). Extracellular vesicles: Structure, function, and potential clinical uses in renal diseases. Braz. J. Med. Biol. Res..

[2] Colombo M., Raposo G., Théry C. (2014). Biogenesis, secretion, and intercellular interactions of exosomes and other extracellular vesicles. Annu. Rev. Cell Dev. Biol..

[3] Welsh J.A., Goberdhan D.C.I., O’Driscoll L., Buzas E.I., Blenkiron C., Bussolati B., Cai H., Di Vizio D., Driedonks T.A.P., Erdbrügger U. (2024). Minimal information for studies of extracellular vesicles (MISEV2023): From basic to advanced approaches. J. Extracell. Vesicles.

[4] Valadi H., Ekström K., Bossios A., Sjöstrand M., Lee J.J., Lötvall J.O. (2007). Exosome-mediated transfer of mRNAs and microRNAs is a novel mechanism of genetic exchange between cells. Nat. Cell Biol..

[5] Kowal J., Tkach M., Théry C. (2014). Biogenesis and secretion of exosomes. Curr. Opin. Cell Biol..

[6] Hombach S., Kretz M. (2016). Non-coding RNAs: Classification, Biology and Functioning. Adv. Exp. Med. Biol..

[7] Peng W.X., Koirala P., Mo Y.Y. (2017). LncRNA-mediated regulation of cell signaling in cancer. Oncogene.

[8] Davidson S.M., Yellon D.M. (2018). Exosomes and cardioprotection—A critical analysis. Mol. Asp. Med..

[9] Statello L., Guo C.J., Chen L.L., Huarte M. (2021). Gene regulation by long non-coding RNAs and its biological functions. Nat. Rev. Mol. Cell Biol..

[10] Luther K.M., Haar L., McGuinness M., Wang Y., Lynch T.L., Phan A., Song Y., Shen Z., Gardner G., Kuffel G. (2018). Exosomal miR-21a-5p mediates cardioprotection by mesenchymal stem cells. J. Mol. Cell. Cardiol..

[11] Momen-Heravi F., Bala S., Bukong T., Szabo G. (2014). Exosome-mediated delivery of functionally active miRNA-155 inhibitor to macrophages. Nanomedicine.

[12] Ying W., Riopel M., Bandyopadhyay G., Dong Y., Birmingham A., Seo J.B., Ofrecio J.M., Wollam J., Hernandez-Carretero A., Fu W. (2017). Adipose Tissue Macrophage-Derived Exosomal miRNAs Can Modulate In Vivo and In Vitro Insulin Sensitivity. Cell.

[13] Nelson H.M., Qu S., Huang L., Shameer M., Corn K.C., Chapman S.N., Luthcke N.L., Schuster S.A., Stamaris T.D., Turnbull L.A. (2024). Transfer of miR-100 and miR-125b increases 3D growth and invasiveness in recipient cancer cells. Extracell. Vesicles Circ. Nucl. Acids.

[14] Feng Y., Huang W., Wani M., Yu X., Ashraf M. (2014). Ischemic preconditioning potentiates the protective effect of stem cells through secretion of exosomes by targeting Mecp2 via miR-22. PLoS ONE.

[15] Fu P., Guo Y., Luo Y., Mak M., Zhang J., Xu W., Qian H., Tao Z. (2023). Visualization of microRNA therapy in cancers delivered by small extracellular vesicles. J. Nanobiotechnol..

[16] Pegtel D.M., Cosmopoulos K., Thorley-Lawson D.A., van Eijndhoven M.A., Hopmans E.S., Lindenberg J.L., de Gruijl T.D., Würdinger T., Middeldorp J.M. (2010). Functional delivery of viral miRNAs via exosomes. Proc. Natl. Acad. Sci. USA.

[17] Buck A.H., Coakley G., Simbari F., McSorley H.J., Quintana J.F., Le Bihan T., Kumar S., Abreu-Goodger C., Lear M., Harcus Y. (2014). Exosomes secreted by nematode parasites transfer small RNAs to mammalian cells and modulate innate immunity. Nat. Commun..

[18] Ong S.G., Lee W.H., Huang M., Dey D., Kodo K., Sanchez-Freire V., Gold J.D., Wu J.C. (2014). Cross talk of combined gene and cell therapy in ischemic heart disease: Role of exosomal microRNA transfer. Circulation.

[19] Nguyen M.A., Karunakaran D., Geoffrion M., Cheng H.S., Tandoc K., Perisic Matic L., Hedin U., Maegdefessel L., Fish J.E., Rayner K.J. (2018). Extracellular Vesicles Secreted by Atherogenic Macrophages Transfer MicroRNA to Inhibit Cell Migration. Arterioscler. Thromb. Vasc. Biol..

[20] He K., Yang T., Yu J., Zang X., Jiang S., Xu S., Liu J., Xu Z., Wang W., Hong S. (2023). microRNAs released to external environments via exosomes regulate inflammation-related gene expression in human bronchial epithelial cells. Front. Immunol..

[21] Okoye I.S., Coomes S.M., Pelly V.S., Czieso S., Papayannopoulos V., Tolmachova T., Seabra M.C., Wilson M.S. (2014). MicroRNA-containing T-regulatory-cell-derived exosomes suppress pathogenic T helper 1 cells. Immunity.

[22] Zomer H.D., Vidane A.S., Gonçalves N.N., Ambrósio C.E. (2015). Mesenchymal and induced pluripotent stem cells: General insights and clinical perspectives. Stem Cells Cloning.

[23] Zhao Y., Sun X., Cao W., Ma J., Sun L., Qian H., Zhu W., Xu W. (2015). Exosomes Derived from Human Umbilical Cord Mesenchymal Stem Cells Relieve Acute Myocardial Ischemic Injury. Stem Cells Int..

[24] Lai R.C., Arslan F., Lee M.M., Sze N.S., Choo A., Chen T.S., Salto-Tellez M., Timmers L., Lee C.N., El Oakley R.M. (2010). Exosome secreted by MSC reduces myocardial ischemia/reperfusion injury. Stem Cell Res..

[25] Barile L., Lionetti V., Cervio E., Matteucci M., Gherghiceanu M., Popescu L.M., Torre T., Siclari F., Moccetti T., Vassalli G. (2014). Extracellular vesicles from human cardiac progenitor cells inhibit cardiomyocyte apoptosis and improve cardiac function after myocardial infarction. Cardiovasc. Res..

[26] Qiu G., Zheng G., Ge M., Wang J., Huang R., Shu Q., Xu J. (2018). Mesenchymal stem cell-derived extracellular vesicles affect disease outcomes via transfer of microRNAs. Stem Cell Res. Ther..

[27] Song Y., Wang B., Zhu X., Hu J., Sun J., Xuan J., Ge Z. (2021). Human umbilical cord blood-derived MSCs exosome attenuate myocardial injury by inhibiting ferroptosis in acute myocardial infarction mice. Cell Biol. Toxicol..

[28] Gao L., Qiu F., Cao H., Li H., Dai G., Ma T., Gong Y., Luo W., Zhu D., Qiu Z. (2023). Therapeutic delivery of microRNA-125a-5p oligonucleotides improves recovery from myocardial ischemia/reperfusion injury in mice and swine. Theranostics.

[29] Mulcahy L.A., Pink R.C., Carter D.R. (2014). Routes and mechanisms of extracellular vesicle uptake. J. Extracell. Vesicles.

[30] Gandek T.B., van der Koog L., Nagelkerke A. (2023). A Comparison of Cellular Uptake Mechanisms, Delivery Efficacy, and Intracellular Fate between Liposomes and Extracellular Vesicles. Adv. Healthc. Mater..

[31] Chen T., Ellman D.G., Fang S., Bak S.T., Nørgård M., Svenningsen P., Andersen D.C. (2024). Transfer of cardiomyocyte-derived extracellular vesicles to neighboring cardiac cells requires tunneling nanotubes during heart development. Theranostics.

[32] Eguchi S., Takefuji M., Sakaguchi T., Ishihama S., Mori Y., Tsuda T., Takikawa T., Yoshida T., Ohashi K., Shimizu Y. (2019). Cardiomyocytes capture stem cell-derived, anti-apoptotic microRNA-214 via clathrin-mediated endocytosis in acute myocardial infarction. J. Biol. Chem..

[33] Krützfeldt J., Kuwajima S., Braich R., Rajeev K.G., Pena J., Tuschl T., Manoharan M., Stoffel M. (2007). Specificity, duplex degradation and subcellular localization of antagomirs. Nucleic Acids Res..

[34] Peister A., Mellad J.A., Larson B.L., Hall B.M., Gibson L.F., Prockop D.J. (2004). Adult stem cells from bone marrow (MSCs) isolated from different strains of inbred mice vary in surface epitopes, rates of proliferation, and differentiation potential. Blood.

[35] Soleimani M., Nadri S. (2009). A protocol for isolation and culture of mesenchymal stem cells from mouse bone marrow. Nat. Protoc..

[36] Zhu H., Guo Z.K., Jiang X.X., Li H., Wang X.Y., Yao H.Y., Zhang Y., Mao N. (2010). A protocol for isolation and culture of mesenchymal stem cells from mouse compact bone. Nat. Protoc..

[37] Théry C., Amigorena S., Raposo G., Clayton A. (2006). Isolation and characterization of exosomes from cell culture supernatants and biological fluids. Curr. Protoc. Cell Biol..

[38] Livak K.J., Schmittgen T.D. (2001). Analysis of relative gene expression data using real-time quantitative PCR and the 2(-Delta Delta C(T)) Method. Methods.

[39] Rio D.C., Ares M., Hannon G.J., Nilsen T.W. (2010). Purification of RNA using TRIzol (TRI reagent). Cold Spring Harb. Protoc..

[40] Lord S.J., Velle K.B., Mullins R.D., Fritz-Laylin L.K. (2020). SuperPlots: Communicating reproducibility and variability in cell biology. J. Cell Biol..

[41] System Biosciences User Manual: Exo-Fect siRNA/miRNA Transfection Kit. https://www.systembio.com/resources/user-manual-exo-fect-sirna-mirna-transfection-kit/.

[42] Lin H.P., Singla B., Ghoshal P., Faulkner J.L., Cherian-Shaw M., O’Connor P.M., She J.X., Belin de Chantemele E.J., Csányi G. (2018). Identification of novel macropinocytosis inhibitors using a rational screen of Food and Drug Administration-approved drugs. Br. J. Pharmacol..

[43] Koivusalo M., Welch C., Hayashi H., Scott C.C., Kim M., Alexander T., Touret N., Hahn K.M., Grinstein S. (2010). Amiloride inhibits macropinocytosis by lowering submembranous pH and preventing Rac1 and Cdc42 signaling. J. Cell Biol..

[44] Kumar S., Wang G., Zheng N., Cheng W., Ouyang K., Lin H., Liao Y., Liu J. (2019). HIMF (Hypoxia-Induced Mitogenic Factor)-IL (Interleukin)-6 Signaling Mediates Cardiomyocyte-Fibroblast Crosstalk to Promote Cardiac Hypertrophy and Fibrosis. Hypertension.

[45] Moreira L.M., Takawale A., Hulsurkar M., Menassa D.A., Antanaviciute A., Lahiri S.K., Mehta N., Evans N., Psarros C., Robinson P. (2020). Paracrine signalling by cardiac calcitonin controls atrial fibrogenesis and arrhythmia. Nature.

[46] Glembotski C.C. (2017). Expanding the Paracrine Hypothesis of Stem Cell-Mediated Repair in the Heart: When the Unconventional Becomes Conventional. Circ. Res..

[47] Gnecchi M., He H., Liang O.D., Melo L.G., Morello F., Mu H., Noiseux N., Zhang L., Pratt R.E., Ingwall J.S. (2005). Paracrine action accounts for marked protection of ischemic heart by Akt-modified mesenchymal stem cells. Nat. Med..

[48] Qiu Z., Liu W., Zhu Q., Ke K., Jin W., Yu S., Yang Z., Li L., Sun X., Ren S. (2022). The Role and Therapeutic Potential of Macropinocytosis in Cancer. Front. Pharmacol..

[49] Jayashankar V., Edinger A.L. (2020). Macropinocytosis confers resistance to therapies targeting cancer anabolism. Nat. Commun..

[50] Cao Y., Wang X., Li Y., Evers M., Zhang H., Chen X. (2019). Extracellular and macropinocytosis internalized ATP work together to induce epithelial-mesenchymal transition and other early metastatic activities in lung cancer. Cancer Cell Int..

[51] Yao W., Rose J.L., Wang W., Seth S., Jiang H., Taguchi A., Liu J., Yan L., Kapoor A., Hou P. (2019). Syndecan 1 is a critical mediator of macropinocytosis in pancreatic cancer. Nature.

[52] Choi D., Montermini L., Meehan B., Lazaris A., Metrakos P., Rak J. (2021). Oncogenic RAS drives the CRAF-dependent extracellular vesicle uptake mechanism coupled with metastasis. J. Extracell. Vesicles.

[53] Dendo K., Yugawa T., Nakahara T., Ohno S.I., Goshima N., Arakawa H., Kiyono T. (2018). Induction of non-apoptotic programmed cell death by oncogenic RAS in human epithelial cells and its suppression by MYC overexpression. Carcinogenesis.

[54] Ritter M., Bresgen N., Kerschbaum H.H. (2021). From Pinocytosis to Methuosis-Fluid Consumption as a Risk Factor for Cell Death. Front. Cell Dev. Biol..

[55] Demasi M., Bechara E.J. (1996). Chlorpromazine stimulatory effect on iron uptake by rat brain synaptosomes. Biochem. Pharmacol..

[56] Hueck I.S., Hollweg H.G., Schmid-Schönbein G.W., Artmann G.M. (2000). Chlorpromazine modulates the morphological macro- and microstructure of endothelial cells. Am. J. Physiol. Cell Physiol..

[57] Fang J., Iwasa K.H. (2007). Effects of chlorpromazine and trinitrophenol on the membrane motor of outer hair cells. Biophys. J..

[58] Kazama I., Ejima Y., Endo Y., Toyama H., Matsubara M., Baba A., Tachi M. (2015). Chlorpromazine-induced changes in membrane micro-architecture inhibit thrombopoiesis in rat megakaryocytes. Biochim. Biophys. Acta.

[59] Cronqvist T., Erlandsson L., Tannetta D., Hansson S.R. (2020). Placental syncytiotrophoblast extracellular vesicles enter primary endothelial cells through clathrin-mediated endocytosis. Placenta.

[60] Ragni E., Banfi F., Barilani M., Cherubini A., Parazzi V., Larghi P., Dolo V., Bollati V., Lazzari L. (2017). Extracellular Vesicle-Shuttled mRNA in Mesenchymal Stem Cell Communication. Stem Cells.

[61] Hinger S.A., Cha D.J., Franklin J.L., Higginbotham J.N., Dou Y., Ping J., Shu L., Prasad N., Levy S., Zhang B. (2018). Diverse Long RNAs Are Differentially Sorted into Extracellular Vesicles Secreted by Colorectal Cancer Cells. Cell Rep..

[62] Usman W.M., Pham T.C., Kwok Y.Y., Vu L.T., Ma V., Peng B., Chan Y.S., Wei L., Chin S.M., Azad A. (2018). Efficient RNA drug delivery using red blood cell extracellular vesicles. Nat. Commun..

[63] Ridder K., Keller S., Dams M., Rupp A.K., Schlaudraff J., Del Turco D., Starmann J., Macas J., Karpova D., Devraj K. (2014). Extracellular vesicle-mediated transfer of genetic information between the hematopoietic system and the brain in response to inflammation. PLoS Biol..

[64] Renault K., Fredy J.W., Renard P.Y., Sabot C. (2018). Covalent Modification of Biomolecules through Maleimide-Based Labeling Strategies. Bioconjug. Chem..

[65] Rodriguez-Caro H., Dragovic R., Shen M., Dombi E., Mounce G., Field K., Meadows J., Turner K., Lunn D., Child T. (2019). Decidualisation of human endometrial stromal cells is enhanced by seminal fluid extracellular vesicles. J. Extracell. Vesicles.

[66] Mittelbrunn M., Gutiérrez-Vázquez C., Villarroya-Beltri C., González S., Sánchez-Cabo F., González M., Bernad A., Sánchez-Madrid F. (2011). Unidirectional transfer of microRNA-loaded exosomes from T cells to antigen-presenting cells. Nat. Commun..

[67] Wang X., Huang W., Liu G., Cai W., Millard R.W., Wang Y., Chang J., Peng T., Fan G.C. (2014). Cardiomyocytes mediate anti-angiogenesis in type 2 diabetic rats through the exosomal transfer of miR-320 into endothelial cells. J. Mol. Cell. Cardiol..

[68] Zhang Z., Xing T., Chen Y., Xiao J. (2018). Exosome-mediated miR-200b promotes colorectal cancer proliferation upon TGF-β1 exposure. Biomed. Pharmacother..

[69] Pan Y., Hui X., Hoo R.L.C., Ye D., Chan C.Y.C., Feng T., Wang Y., Lam K.S.L., Xu A. (2019). Adipocyte-secreted exosomal microRNA-34a inhibits M2 macrophage polarization to promote obesity-induced adipose inflammation. J. Clin. Investig..

[70] Guo X., Qiu W., Liu Q., Qian M., Wang S., Zhang Z., Gao X., Chen Z., Xue H., Li G. (2018). Immunosuppressive effects of hypoxia-induced glioma exosomes through myeloid-derived suppressor cells via the miR-10a/Rora and miR-21/Pten Pathways. Oncogene.

[71] Geng T., Ding L., Liu M., Zou X., Gu Z., Lin H., Sun L. (2025). Preservation of extracellular vesicles for drug delivery: A comparative evaluation of storage buffers. J. Drug Deliv. Sci. Technol..

[72] Liu J.F., Xia J., Rich J., Zhao S., Yang K., Lu B., Chen Y., Ye T.W., Huang T.J. (2025). An Acoustofluidic Device for Sample Preparation and Detection of Small Extracellular Vesicles. Cyborg Bionic Syst..

[73] Kleinman D., Iqbal S., Ghosh A.K., Ogle S.D., Kaja S., Mitchnick M., Hakkarainen J.J. (2024). PLL-g-PEG Polymer Inhibits Antibody-Drug Conjugate Uptake into Human Corneal Epithelial Cells In Vitro. J. Ocul. Pharmacol. Ther..

[74] Dabre Z., Krupa E.G., Pasupuleti B., Bacellar-Galdino M., Troughton L.D., Mun C., Hernandez E., Moon H.G., Flavin M.T., Kaja S. (2026). Ocular Tissue Distribution of Ocular Surface Immunoglobulin Following Topical and Subconjunctival Administration: A Novel Strategy for Mitigation of Antibody-Drug Conjugate-Associated Ocular Toxicity. J. Ocul. Pharmacol. Ther..

