# Supplementary material for: MSC EVs Deliver miRNAs into Cardiomyocytes via EIPA-Sensitive and Clathrin-Mediated Endocytosis
Source: Biomedicines. 2026 Sep 5;14(9):2002. doi: 10.3390/biomedicines14092002 (PMC13604255; doi:10.3390/biomedicines14092002)
Supplement: Supplementary file 1 [file biomedicines-14-02002-s001.zip › biomedicines-4506012-supplementary-final.pdf]

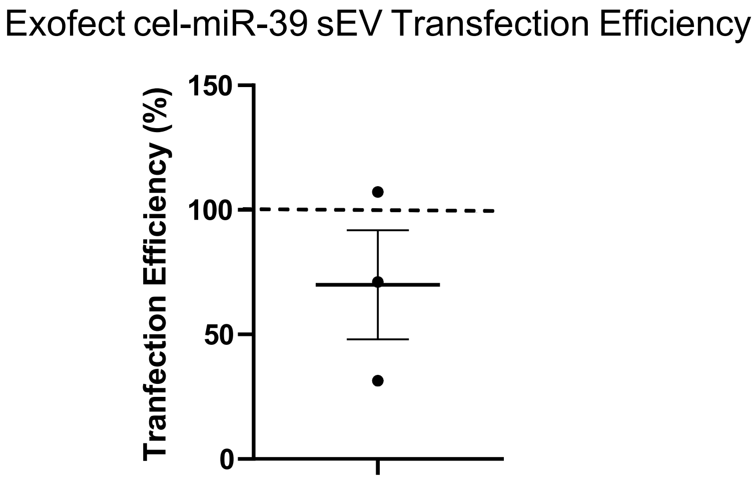

Supplementary Figure S1. Relative recovery of cel-miR-39-3p after Exo-Fect/ExoQuick processing. cel-miR-39-3p was measured by qRT-PCR and normalized to miR-21a-5p using the ΔΔCT method. Relative transfection efficiency was calculated as the percentage of post-transfection cel-miR-39-3p signal compared with the pre-transfection comparator. Data are presented as mean ± SEM; n = 3.

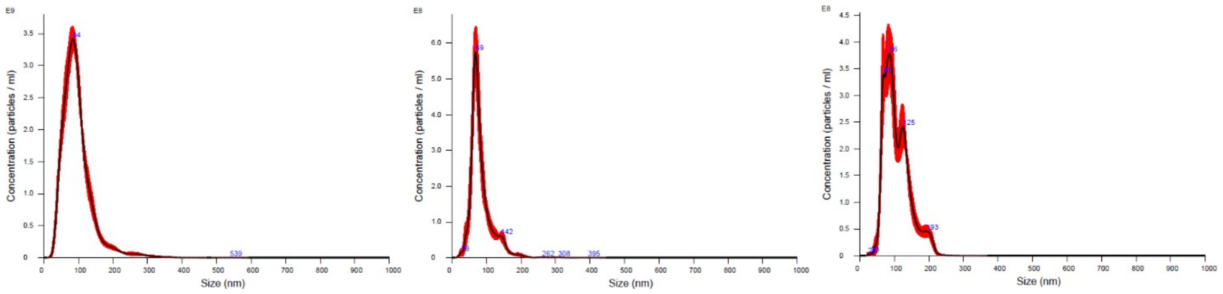

Supplementary Figure S2. Exo-Fect and ExoQuick treatment does not significantly alter MSC EV size distribution. NanoSight traces of MSC EVs before (left) and after being transfected with cel-miR-39-3p and Exo-Fect reagent (mid), and isolated with ExoQuick reagent (right).

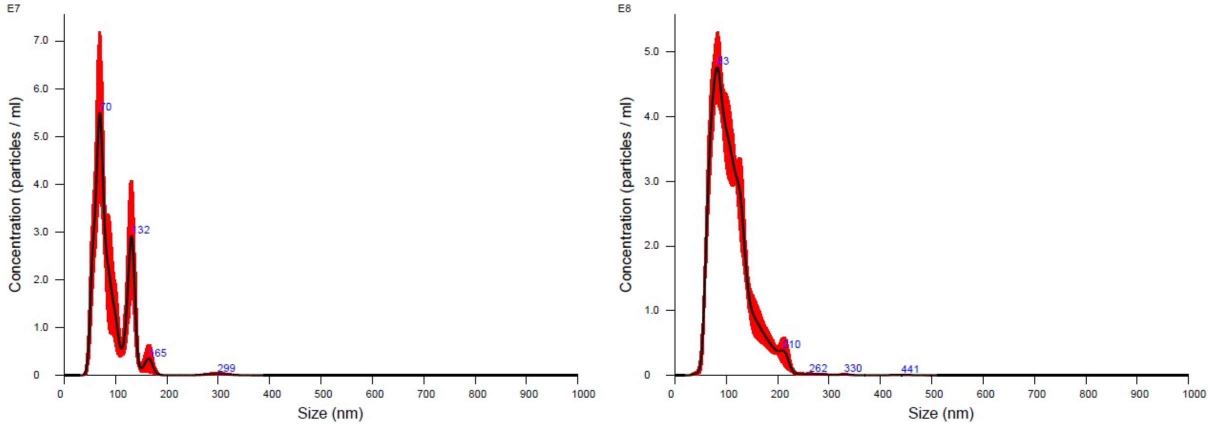

Supplementary Figure S3. PBS control forms nanoparticles following Exo-Fect treatment. NanoSight traces from same volume of PBS (left, conc. 2.29×10^{9} particles/mL) and MSC EVs (right, conc. 3.42×10^{10} particles/mL) after being transfected with cel-miR-39-3p and Exo-Fect reagent, though to a much less amount.
